# Active Double-Layered Films Enriched with AgNPs in Great Water Dock Root and Pu-Erh Extracts

**DOI:** 10.3390/ma14226925

**Published:** 2021-11-16

**Authors:** Ewelina Jamróz, Agnieszka Cabaj, Lesław Juszczak, Joanna Tkaczewska, Małgorzata Zimowska, Agnieszka Cholewa-Wójcik, Paweł Krzyściak, Pavel Kopel

**Affiliations:** 1 Department of Chemistry, University of Agriculture, ul. Balicka 122, 30-149 Kraków, Poland; ewelina.jamroz@urk.edu.pl; 2 Department of Food Analysis and Evaluation of Food Quality, University of Agriculture, ul. Balicka 122, 30-149 Krakow, Poland; agnieszka.grabeus@urk.edu.pl (A.C.); rrjuszcz@cyf-kr.edu.pl (L.J.); 3 Department of Animal Product Technology, Faculty of Food Technology, University of Agriculture, ul. Balicka 122, 30-149 Kraków, Poland; tkaczewska@gmail.com; 4 Jerzy Haber Institute of Catalysis and Surface Chemistry, Polish Academy of Sciences, ul. Niezapominajek 8, 30-239 Kraków, Poland; malgorzata.zimowska@ikifp.edu.pl; 5 Department of Product Packaging, Cracow University of Economics, ul. Rakowicka 27, 30-510 Kraków, Poland; cholewaa@uek.krakow.pl; 6 Department of Infections Control and Mycology, Jagiellonian University Medical College, ul. Czysta 18, 31-121 Kraków, Poland; pawel.krzysciak@uj.edu.pl; 7 Department of Inorganic Chemistry, Faculty of Science, Palacky University, 17. Listopadu 12, CZ-771 46 Olomouc, Czech Republic

**Keywords:** furcellaran, gelatin hydrolysate, chitosan, double-layered films, silver nanoparticles

## Abstract

A novel, eco-friendly, and biocompatible method was applied to form silver nanoparticles (AgNPs) in great water dock (*Lapathi radix*) (KB) and pu-erh (*Camellia sinensis*) (PE) extracts. The surface plasma resonance peak of green synthesized AgNPs at 451.8 nm for AgNPs+KB and 440.8 nm for AgNPs+PE was observed via spectral analysis of UV absorbance. In this study, double-layered biopolymer films (FUR/CHIT+HGEL) with AgNPs incorporated in KB solution (AgNPs+KB) and AgNPs in PE solution (AgNPs+PE), were successfully prepared using the casting method. The SEM, XRD, zeta potential and size analyses confirmed the presence of AgNP in the films. The addition of AgNPs in plant extracts improved antimicrobial and antioxidant activity and thermal stability, whereas WVTR experienced a decrease. The nanocomposite films’ orange-brown colour may aid in the protection of food products against UV rays. The composite films demonstrated antibacterial activity against food-borne pathogens and may offer potential in food packaging applications.

## 1. Introduction

There are several types of nanoparticle syntheses using physical, chemical and biological methods. The use of physical and chemical methods entails high energy consumption and is often associated with the use of toxic substances. Biological methods are more cost-effective and non-toxic to the environment, and using plant extracts for the synthesis of nanoparticles offers many advantages over other approaches. Plants are easily available and widespread; they offer low production costs and are a source of several metabolites (bioactive phytochemicals) [1]. The use of plant extracts in the synthesis of AgNPs is a pro-environmental solution, because they are rich in reducing and stabilising substances, such as enzymes, polyphenols and proteins [2]. Thanks to this solution, toxic compounds, such as sodium borohydride (NaBH_4_), sodium citrate, and ascorbate that are not consumed by consumers are not used; thus, their use in the preservation of food products is limited. The leaves, stems, roots, fruits, and seeds of plants can be effectively used for the production of nanoparticles because they actively reduce metal ions and act as protective agents and stabilisers for NP. In particular, the biosynthesis of AgNPs using plant extracts deserves attention in its application. Phytochemicals contained in the plant feature antioxidant and reducing properties and therefore act on silver particles by creating nanoparticles (redox reaction) [1].

Among the various metallic nanoparticles, silver nanoparticles (AgNPs) are stable, and they are widely used in cosmetics, nanomedicine and food processing, as well as being an effective antimicrobial agent. There are many studies in which the properties of AgNPs obtained from plant extracts are investigated [3,4,5,6]. However, there are no published data describing the synthesis of AgNPs in pu-erh green tea leaf and great water dock root extracts.

Great water dock (*Lapathi radix*) is used worldwide in conventional medicine for treating a wide range of ailments caused by various microorganisms (e.g., bacterial skin disorders, dysentery, enteritis) [7]. Pu-erh green tea (*Camellia Sinensis* var. *assamica*) is a post-fermentation tea, which was originally produced in China’s Yunnan Province for about 1700 years, and is used for health purposes in Asian countries [8]. Because various microorganisms take part in the post-fermentation process, pu-erh tea contains very complex ingredients demonstrating rich interactions with the gut microbiome [9].

Biopolymer-based films feature many disadvantages that must be overcome, including: poor mechanical strength, high solubility, and high gas permeability. Biopolymer, double- or multilayer films can overcome these disadvantages, as each of the layers adheres to the others and can be enriched by different active substances [10,11]. Zhang, et al. [12] added the antibacterial ingredient cinnamon essential oil to the multilayer films based on chitosan and sodium alginate. The obtained results confirmed that the multilayer film showed a more sustained release of the antibacterial agent and also caused greater oil retention in the multiphase system than the monolayer film. Double-layered films based on furcellaran, CMC, and gelatin hydrolysate enriched with lingonberry extract demonstrated very high thermal stability as well as antioxidant and antimicrobial activity [13]. Zhou et al. [11] produced a double-layered indicator film, where the indicator layer was made of carrageenan, curcumin and/or anthocyanin, and the emulsified layer made of konjac glucomannan and camellia oil. The resulting double-layered indicator film demonstrated a distinct colour change when monitoring the freshness of the chicken meat. Multilayer films based on cationic guar gum and anionic cellulose nanofibrils were obtained using the layer-by-layer casting method. The obtained films featured very good gas and oil barrier properties, and were resistant to various organic solvents [14].

Furcellaran is a negatively charged polysaccharide that is extracted from the red algae, *Furcellaria lumbricalis*. This sulfate polysaccharide may act as a component in biopolymer films and can be used as one of the coatings in the preparation of nanocapsules [15]. Chitosan consists of (1,4)-linked 2-amino-deoxy-b-d-glucan and is a well-known film-forming material with good mechanical, biological (particularly, antimicrobial and antioxidant), and chemical properties [16]. Gelatin hydrolysates, which are obtained from post-industrial fish industry waste, become a good active ingredient which, due to their very good antioxidant activity, can act as a functional food component and ingredient in biopolymer films [17].

There is a lack of research data describing the effect of AgNP plant extracts on biopolymer-based double-layered film properties. Therefore, the study objective was to design an innovative approach towards the green biosynthesis of AgNP while utilising the aqueous extract of great water dock (*Lapathi radix*) (AgNPs+KB) and pu-erh (*Camellia sinensis*) (AgNPs+PE). Another goal of this study was to evaluate the effects of AgNPs+KB and AgNPs+PE extracts on the structural, functional, and bioactive properties of double-layered films based on furcellaran, chitosan, and carp skin gelatin hydrolysates (FUR/CHIT+HGEL). The study concludes that it is possible to develop double-layered films on the basis of furcellaran and chitosan with gelatin hydrolysate enriched with great water dock and pu-erh extracts.

## 2. Materials and Methods

### 2.1. Materials

The furcellaran (FUR) (type 7000) was purchased from Est-Agar AS (Karla village, Estonia). The composition related to the chemical of FUR (M_w_ 2.951 × 10^5^) was: 79.61% carbohydrates, 1.18% protein, and 0.24% fat. The chitosan (CHIT) (molecular weight ~890 kDa, CAS number 9012-76-4, ≥90% deacetylation, 100–300 mPa∙s viscosity; particle size ≤ 100 mesh) was acquired from POL-AURA (Zabrze, Poland). The carp skin gelatin hydrolysates were procured according to the method previously described by Tkaczewska et al. [18]. The great water dock root (*Lapathi radix*) root was obtained from the FLOS company (Mokrsko, Poland), while the pu-erh tea was manufactured in Yunnan (China). The chemical reagents were applied as received, not being subjected to further purification in any way.

### 2.2. Preparation of Plant Extracts

The Pu-eh leaves (10 g) or great water dock root (10 g) were added to distilled water (100 mL) and heated to 80 °C for 30 min with the use of a magnetic stirrer. The aqueous extract was filtered three times and cooled at room temperature (~25 °C).

### 2.3. Silver Nanoparticle Synthesis (AgNPs)

For the production of the AgNPs + plant extract solution, we adopted the method described by [19], with modifications. From the prepared solution, 5 mL of plant extract were taken and 15 mL of distilled water was added. The green synthesis of AgNPs was performed with the addition of 0.5 mL of AgNO_3_ (0.1 M) to the prepared solution. In the case of the pu-erh (PE) solution, the colour change was observed after 30 min; however, with regard to great water dock (KB), the reaction took place after approx. 3 h.

### 2.4. Double-Layered Film Preparation

In order to prepare the double-layered film, glycerol (1% *w*/*w*) was added as a plasticiser to a 1% solution of furcellaran. The obtained solution was heated to a temperature of 100 °C and then continuously stirred at 500 rpm until it completely dissolved. Next, the temperature of the FUR solution was lowered to 60 °C and various contents of specific plant extracts enriched with AgNP solution (5%, 10% and 15% *v*/*v*) were added.

The solutions prepared in this way were poured into Petri dishes and allowed to gel for 30 min. At this point, glycerol (1% *w*/*w*) and 0.25 g of gelatin hydrolysate (HGEL) were added to the prepared 1% chitosan solution (CHIT) and further stirred for a duration of 30 min. The solution prepared in this manner was poured onto the previously prepared mould with the first furcellaran layer and was allowed to dry in the frame hood switched on for 1 day. The films were marked as follows: films with great water dock root extract enriched in AgNPs—FUR+AgNPs5KB/CHIT+HGEL; FUR+AgNPs10KB/CHIT+HGEL; FUR+AgNPs15KB/CHIT+HGEL and films with pu-erh extract enriched in AgNPs—FUR+AgNPs5PE/CHIT+HGEL; FUR+AgNPs10PE/CHIT+HGEL, and FUR+AgNPs15PE/CHIT+HGEL.

### 2.5. UV-Vis Spectroscopy Analysis

The UV-Vis analysis was conducted by applying a UV-5500 spectrophotometer (UV 5500 Metash), while the absorbance spectrum was noted between 200 nm and 800 nm to confirm the formation of AgNP in the extracts, as well as the presence of AgNPs in the tested films.

### 2.6. Particle Size and Zeta Potential

The particle size and zeta potential of the nanoparticles were assessed at 25 °C via dynamic light scattering (DLS) (Zetasizer Ultra Red, Malvern Instruments Ltd., Worcestershire, UK).

### 2.7. X-ray Diffraction Patterns (XRD)

The XRD analysis was conducted via the X’Pert PRO (PANalytical B.V.) diffractometer, which was operated at 40 kV and 30 mA using Ni-filtered Cu Kα radiation. All of the recorded patterns were between 5° and 72°, with a 0.05° step size. Furthermore, the samples were prepared by means of oriented thin films, the polymer composite suspension being dried on a glass slide.

### 2.8. Scanning Electron Microscopy (SEM)

The morphology of the double-layered films was assessed using a JEOL JSM–7500F Field Emission Scanning Electron Microscope, model: JEOL JSM–7500F, manufactured by JEOL Ltd. Tokyo, JAPAN. This microscope contains the Retractable Backscattered-Electron detector (RBEI), a Transmission Electron detector (TED) and an EDS (energy dispersive spectra) detection system of distinctive X-ray radiation, i.e., INCA PentaFetx3 EDS system.

### 2.9. Colour Parameters

The colour on the film surface was evaluated via the method of reflection, incorporating a Color i5 spectrometer (X-Rite, Grand Rapids MI, USA, illuminant D65). The results of the evaluation were presented in the following manner: L* (lightness), a* (red-green) and b* (yellow-blue). Additionally, the following formula was applied to calculate the total value of difference in colour (ΔE):

∆E = √((∆a)^2^ + (∆b)^2^ + (∆L)^2^) (1)

where ΔE is the total colour difference value, considering the FUR/CHIT+HGEL film as a standard, and FUR/CHIT+HGEL films, including extracts + AgNPs.

### 2.10. Water Vapour Transmission Rate (WVTR)

The WVTR test was measured based on the ISO 2528:2017 standard (sheet materials, determination of water vapour transmission rate (WVTR), gravimetric (dish) method). Silica gel was placed into a glass vessel until filled. The vessel was further covered by the film under analysis and then placed in a chamber with the possibility of regulating the microclimate (conditions: C-temperature at 25 °C, 75% relative humidity). Subsequently, we weighed the vessel at specified points of time. A gain in weight was implemented to assess WVTR. The specimens for testing were prepared in a normative manner and subjected to air conditioning. The WVTR was estimated as follows:

WVTR (g/m^2^·d) = 240·(water weight/surface penetration)·24 (2)

### 2.11. Determination of Contact Angle

The water contact angle (WCA), relative to the water found in the film under evaluation, was determined using the sessile drop technique, implementing a system for carrying out video-based measurements of the contact angles (OCA, Dataphysics, Filderstadt, Germany). This was performed at room temperature (~23 °C). A droplet (8 µL) of deionised water was gradually dripped onto the surface of the film using a micro-injector. The image was acquired on this basis. The measurements were carried out on five samples.

### 2.12. Thermal Properties

About 2 mg of the sampled film was weighed, sealed in aluminum pans, and then heated from a temperature of 30 °C to 300 °C increasing the temperature by 5 °C/min. The empty pan was treated as a reference. The tests were conducted using a DSC 204F1 Phoenix differential scanning calorimeter (Netzsch, Selb, Germany) and the factors regarding the thermal transition under observation were assessed using the Proteus Analysis software (Netzsch, Selb, Germany). The measurements were carried out on three samples.

### 2.13. Antioxidant Activity Measured via FRAP Method

The preparation of the film extracts (10 mg/mL) was carried out by adding the films in the amount of 100 mg to 10 mL of distilled water, and then heating the mixture to 45 °C. The tubes, including the film extracts, were placed into a water bath (at 50 °C) and shaken for 10 min until the films completely dissolved. The extracts prepared in this manner were used for FRAP (Ferric Reducing Antioxidant Power) assay. The FRAP solution was freshly prepared, just before the analysis. It comprised an acetate buffer (pH 3.6), a ferric chloride solution (20 mM), and a 2,4,6-tripyridyl-s-triazine solution (10 mM TPTZ in 40 mM HCl) at a 10:1:1 (*v*/*v*/*v*) ratio, respectively. First, the FRAP solution was incubated in the dark at a temperature of 37 °C for 30 min. Next, it was mixed after adding the film extract, applied at a 0.4:3.6 (*v*/*v*) ratio. The solution was once again incubated in the dark (for a duration of 10 min) at a temperature of 37 °C. Subsequently, the solution absorbance was evaluated at 593 nm, applying the Helios Gamma UV-1601 spectrophotometer (Thermo Fisher Scientific, Waltham, MA, USA).

### 2.14. Antimicrobial Measurements

Antimicrobial research on films was described in a previous study [20]. The antimicrobial influence against *Staphylococcus aureus* ATCC 25923, *Escherichia coli* ATCC 25922, *Pseudomonas aeruginosa* ATCC 27869, *Enterococcus faecalis* ATCC 29212, *Salmonella enterica* BAA664, and the fungi, *Candida krusei* ATCC 6258, was evaluated via an assay of agar disc diffusion. The films were pieced (1 cm × 1 cm) and placed on agar plates (Muller Hinton 2 in the case of bacteria, and Sabouraud Glucoe Agar in the case of yeast). Next, each 10 mL liquefied (40–50 °C) medium was inoculated with a standard microorganism suspension (1–5 × 106 CFU mL^−1^ and 1–5 × 104 CFU mL^−1^, bacteria and yeasts respectively). Then poured onto agar plates containing the tested films and were subsequently allowed to solidify, before being incubated at a temperature of 37 °C for a duration of 24 h. The measurements were performed visually, comparing the microorganism growth in the area under and around the pieces of film.

### 2.15. Statistical Analysis

For assessing the significance of the differences between the average values, the data were analysed with regard to variance. The significance of the differences between the means was established via Fisher’s LSD test; the level of statistical significance assumed as 0.05. Calculations were carried out via Statistica 12.0 (StatSoft Inc., Tulsa, OK, USA).

## 3. Results and Discussion

### 3.1. UV-Visible Spectral Study

**Figure 1:**
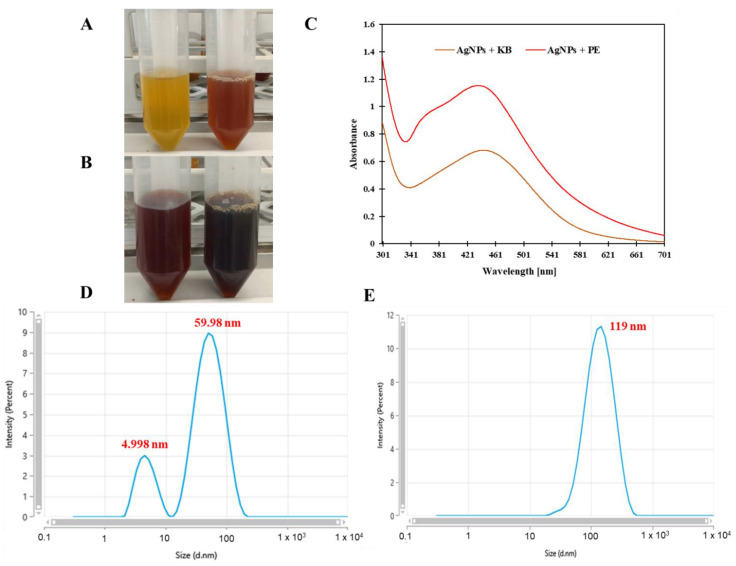
Discoloration of solutions during AgNPs preparation using (**A**) water dock extract; (**B**) pu-erh extract; (**C**) ultraviolet-visible absorption spectrum of AgNPs-KB and AgNPs-PE extracts. (**D**) size of AgNPs-PE extract; (**E**) size of AgNPs-KB extract.

The water extracts of water dock and pu-erh made it possible to obtain opaque solutions of light brown and beige colour. The preparation of the silver nanoparticles using AgNO_3_ made the solutions appear cloudy and dark, indicating the formation of AgNPs in the extracts (Figure 1A,B). The colour changes in the solutions can be attributed to the rise in the number of silver nanoparticles in the reaction mixture [3]. The reduction and wetting of photochemical compounds in the extracts of great water dock and pu-erh caused the occurrence of a surface plasmon resonance (SPR) peak. This formation of AgNPs is assured with this SPR peak in the UV spectrum [5]. The UV-Vis study allowed us to confirm the presence of AgNPs, as the absorption spectrum was detected, totalling 451.8 nm for AgNPs+KB and 440.8 nm for AgNPs+PE, respectively (Figure 1C). The absorption peak in the visible light region (400–500 nm) is evidence of SPR of the AgNPs presence, showing that the Ag ions of the AgNO_3_ solution changed into a metallic form of AgNP. These results are in line with those obtained in previous studies [1,4,21]. The fact that the silver nanoparticles are well dispersed without aggregation in the aqueous medium is evidenced by the fact that the UV-Vis band of colloidal silver was around 430–450 nm [3]. Moreover, the presence of a strong SPR peak in this region has been documented for various AgNPs ranging in size from 5 to 100 nm [22].

### 3.2. Dynamic Light Scattering Analysis

The particle size was measured using the dynamic light scattering technique. The calculated average size of the nanoparticles for KB was 119 nm. In the case of PE, silver nanoparticles in the size of 4.998 and 59.98 nm were recorded. It was also found that the zeta potential of the synthesised silver nanoparticles totalled −28.12 mV (PE) and −29.48 mV (KB). According to the applicable research items, the obtained solutions with AgNPs can be considered moderately stable (±20–30 mV) [1]. The obtained values make it possible to confirm the effective stability of AgNPs in plant extracts. Biofunctional components in aqueous roots and leaf extracts cause the potential of the synthesised green AgNPs to have a negative value [5]. The negative charge and stability can be attributed to the plant components which coat the surface of AgNPs, where the negative charge is similarly derived from the carboxyl group obtained after the hydroxyl reaction [23].

### 3.3. Characterization of Double-Layered Films with AgNPs-Great Water Dock Root and AgNPs-Pu-Erh Extracts

**Figure 2:**
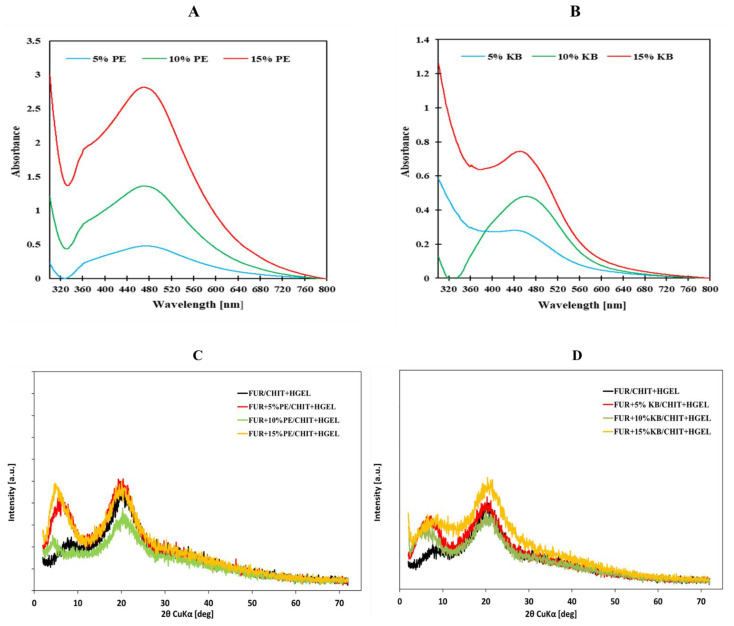
UV-Vis spectra of double-layered films with AgNPs-PE (**A**), AgNPs-KB and XRD patterns of double-layered films (**B**), AgNPs-PE (**C**), and AgNPs-KB (**D**).

The next step was the incorporation of the AgNP plant extracts into the double-layered biopolymer films. The double-layered films consisted of first-layer-furcellaran (FUR) and second-layer-chitosan + gelatin hydrolysate (CHIT+HGEL). Plant extracts in various concentrations were added to the FUR layer in the double-layered films. Furcellaran plays an important role as a reducing and stabilising agent for Ag^+^ [24]. In this study, it was used as a biopolymer matrix. The UV-Vis spectra of the films with the addition of AgNPs are presented in Figure 2A,B. As shown in the Figures, the films enriched with AgNPs-PE and AgNPs-KB solutions showed a better ability to absorb UV light within the wavelength range of 380–480 nm.

The XRD technique was used to determine the crystal structure of the obtained nanocomposites (Figure 2C,D). The analysis of XRD patterns revealed the presence of Bragg’s reflection at c.a. 2Θ = 20.35°, which was attributed to furcellaran with similar semi-amorphous ordering of the structure in the samples. The small reflection at 11° of 2Θ with some contribution in the broad peak at 21° confirmed the presence of chitosan in all the synthesized samples [25,26].

The addition of AgNP-PE and AgNP-KB dopants to the nanocomposites did not change the position of the wide peak observed within the range of 15 to 25° of 2θ, indicating the constant amorphous nature of the films [27]. However, it also contributed to the appearance of the reflection at ca. 5° of 2θ, revealing the presence of the extracts in the double-layered films [28]. The FUR+AgNPs PE/CHIT+HGEL nanocomposites demonstrated weak diffraction peaks at ~38°, ~44°, and ~64°, which corresponded to (111), (200), and (220) for silver metallic reflections and represented the cubic crystal of the silver structure [29,30]. The diffraction peaks related to the metallic silver structure in FUR+AgNPs- KB/CHIT+HGEL were hidden in the background around ~38°, ~44°, and ~64° of 2θ, which revealed the low dimensions of silver crystallites, below the detection threshold in the XRDmethod.

### 3.4. SEM Analysis

**Figure 3:**
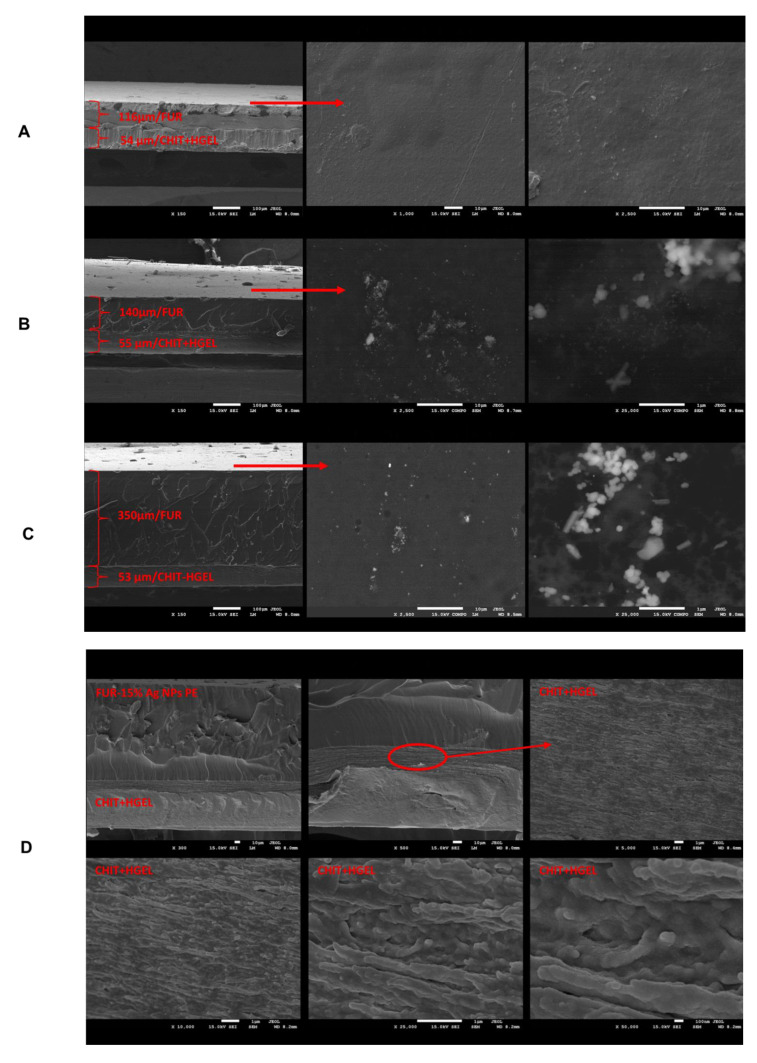

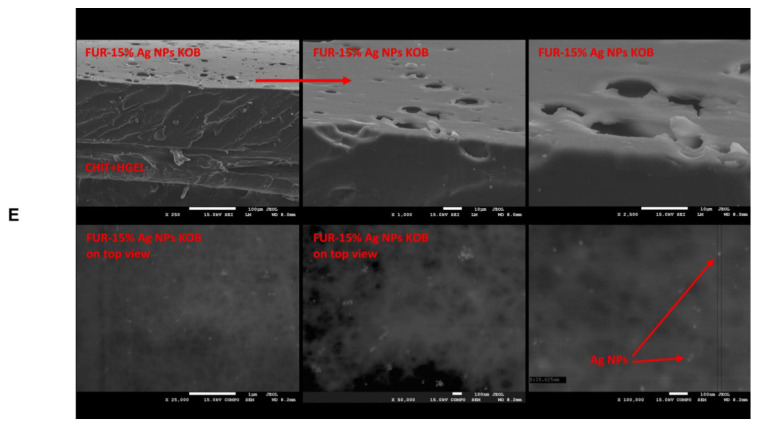
SEM images of double-layered films: (**A**) FUR/CHIT+HGEL; (**B**) FUR+5AgNPs-PE/CHIT+HGEL; (**C**,**D**) FUR+15AgNPs-PE/CHIT+HGEL; (**E**) FUR+15AgNPs-KB/CHIT+HGEL.

To gain an insight into the microstructure of the nanocomposite films, SEM microscopy was employed. Figure 3 shows that the film consisted of two layers: an upper layer, attributed to the FUR component, and a lower layer, attributed to the CHIT+HGEL film.

The furcellaran layer in the FUR/CHIT+HGEL film featured a smooth structure, which was also confirmed by our previous research on this type of film [24]. The second layer in this system featured a more sheet/fibrous-like structure, derived from the mixture of chitosan with gelatin hydrolysate (Figure 3A). The characteristic appearance of the gelatin hydrolysate has been observed before [17]. The BSE images of the nanocomposites reveal the formation of Ag nanoparticles (visible as the brighter areas and tiny spots) with an average dimension about 20 nm in the FUR layer. The distribution of Ag nanocrystallites was not quite homogeneous and some aggregations of AgNPs nanoparticles appeared according to the plant extract used (Figure 3D,E). The same results were observed by Kumar et al. [21]. As an example, the FUR+AgNPs PE/CHIT+HGEL films with the distribution of AgNPs where particles were not evenly dispersed in the bulk of the nanocomposite films, are presented in Figure 3B–D. On the other hand, FUR+AgNPs KB/CHIT+HGEL films exhibited the presence of nanoparticles with an exactly uniform distribution as is depicted in Figure 3E. The plant solutions with AgNPs affected the structure of the FUR/CHIT+HGEL in the double-layered film. In the case of AgNP production by the in situ method on the FUR matrix, an even distribution of nanoparticles was observed [24]. In this instance, the distribution of the nanoparticles may have depended on the miscibility of the FUR matrix with the plant extract, as well as the type of plant extract used for the synthesis of the AgNPs.

### 3.5. Physical Properties of Tested Films

The control films (FUR/CHIT+HGEL) were transparent and homogeneous, while the addition of AgNPs enriching the plant extracts made the films less transparent, obtaining a brown colour of varying intensity depending on the type of extract used and its concentration (Table 1). After incorporating the extracts into the films, the L* value dropped significantly due to the darkening of the films. However, the films with the addition of AgNPs-PE showed a greater decrease in the value of this parameter. The a* and b* values increased significantly, which indicates a tendency towards reddening and yellowing. The obtained results are consistent with the visual observations (Table 1).

Along with the increase in the concentration of the extracts enriched with AgNPs, the T_m_ values increased; however, these values for the film with the addition of AgNPs+PE were much higher, which may indicate that such an addition causes greater changes in the structure of the film, thus increasing thermal stability. The inclusion of AgNPs in the furcellaran matrix may result in improved thermal insulation or additional resistance to the transfer of volatile products produced by temperature changes during the storage of the films [19].

The addition of AgNP plant extracts increased the WVTR value of the double-layered films (Table 1). These values are dependent on the hydrophobicity–hydrophilicity ratio of the film, according to which a film with a more hydrophilic nature may exhibit higher WVTR values by increasing the degree of hydrogen bonding [31,32]. The production of AgNPs by the in situ method on the FUR matrix decreased the WVTR value of the FUR films [24]. In this case, two-stage film production occurred. In the first stage, AgNPs were produced in the plant extracts, while the extracts prepared in this way were added to the first layer of the film-forming matrix. Moreover, the even distribution of nanoparticles from the KB extract in the films (Figure 3) may have resulted in lower WVTR values.

The water contact angle (WCA) of the obtained films was tested to determine their hydrophilicity/hydrophobicity (Table 1). The WCA of the first layer (FUR) was 86.34, indicating that the surface was hydrophobic. The addition of AgNPs increased the hydrophobicity of nanocomposite films, which was related to the hydrophobic metallic nature of the silver AgNPs [33]. Moreover, the addition of AgNPs to the furcellaran layer influenced the second layer of nanocomposites (CHIT+HGEL), reducing the WCA value. The preservation of the furcellaran layer in this system is significant. Furcellaran itself features hydrophilic properties, while in a double-layered system, it is characterised by hydrophobicity. The decrease in WCA value may have been due to the increase in the film’s roughness after the introduction of the metallic AgNPs. Shankar et al. [34] reached similar conclusions after adding AgNPs made from *Caesalpinia minosoides* Lamk. extract to pectin films.

### 3.6. Antioxidant Activity of the Tested Films

The FUR/CHIT+HGEL film demonstrated moderate antioxidant activity due to the presence of the carp skin gelatin hydrolysate. Our previous results clearly proved that the hydrolysate features a good ferric-reducing ability [35,36]. The obtained results indicate that the antioxidant activity of the double-layered film increased with the increase in the concentration of the AgNPs extracts, which may have been due to the presence of bioreductive functional groups of molecules adhering to the surface of nanoparticles [37]. Silver ions can act as antioxidants by transferring a single electron and a hydrogen atom [38]. Furthermore, the antioxidant activity of water dock root extract and the puh-er extract is due to the presence of such components as condensed tannins, phenols, and flavonoids [39,40]. According to Pereira de Abreu, et al. [41], the increase in the antioxidant concentration in the film generates a greater protective effect on secondary oxidation in model food products. It may be assumed that the films with the highest concentration of AgNPs could be effective at inhibiting lipid oxidation in perishable food products. However, for this to be confirmed, further research should be conducted.

### 3.7. Antimicrobial Activity of the Tested Films

Considering that the developed materials can act as active packaging, several strains of bacteria and a fungus were selected (Table 2).

Among the Gram-negative bacteria, *Escherichia coli* was assessed as an indicator of deficient hygiene practices, *Pseudomonas aeruginosa* as an indicator of general hygiene in the production area, and *Salmonella* spp. as the obligatory quality criterion in the absence of this micro-organism among foodstuffs. *S. aureus* spp., a Gram-positive bacteria indicating inappropriate food handling due to deficient operator hygiene, was also tested [42]. The obtained nanocomposite films showed antibacterial activity, which may have been related to the release of silver ions that attached to the surface of the cells and proteins responsible for metabolic processes [21]. The inhibition of bacterial growth is due to the interaction between the additionally charged silver ions and negatively charged particles containing sulfur or phosphorus in the bacteria, and this leads to structural changes that result in cell death [2,43]. Kumar et al. [21], who produced corn starch and silver nanoparticles+guava leaves extract, reached similar conclusions. Moreover, AgNPs, due to their smaller size, penetrate bacterial cells more easily [33]. It is also believed that the antimicrobial activity of AgNPs depends on the surface area of the nanoparticles, which are likely to act as reservoirs for the release of Ag^+^ ions [44]. Another probable mechanism underlying the antimicrobial action of AgNPs is related to damage to the membrane by free radicals from the surface of nanoparticles, thereby causing a significant increase in membrane permeability and subsequent cell death [45,46]. Silver nanoparticles show stronger activity against Gram-negative bacteria than against Gram-positive bacteria [46,47]. This behavior may be due, in part, to differences in the structure of the bacterial cell wall. Gram-positive bacteria feature a thick peptidoglycan layer (20–80 nm), creating a complex structure that is difficult to penetrate. By contrast, Gram-negative bacteria are covered with a thin layer of peptidoglycan (7–8 nm), which facilitates the penetration of AgNPs inside the cell. In the results obtained in this study, it can be seen that the largest zone of inhibition was in *p. aeruginosa*, which is a Gram-negative bacteria. However, it was also observed that similar sizes of the zones of inhibition were present in *S. aureus* and *E. coli*. In conclusion, AgNPs produced with the use of medicinal plant extracts can be a very effective bactericide embedded in the matrix of double-layered films that are used in biomedicine and related fields. However, it is necessary to conduct research on the cytotoxicity of the material and the migration of AgNPs, since only this type of research will make it possible to indicate the potential direction of the use of double-layered films.

## 4. Conclusions

The present study demonstrates the procedure of silver nanoparticle (AgNPs) formation in great water dock root (*Lapathi radix*) (AgNPs-KB) and pu-erh (*Camellia sinensis*) (AgNPs-PE) extracts. The phenolic compounds are capable of forming and stabilising AgNPs. The synthesis of AgNPs in plant extracts was carried out and the mixtures demonstrated typical colours, as well as UV-visible spectra, which was particularly associated with AgNPs. These plant extracts with AgNPs were successfully incorporated into the first layer (FUR) of the double-layered films. The results of the UV-VIS Spectroscopy, SEM, and size evaluation revealed that the AgNPs featured an average size below 100 nm. Due to the addition of AgNP fillers to the double-layered films, the thermal properties, water sensitivity, and antioxidant and antimicrobial activity improved. The prepared films demonstrated highly effective antimicrobial activity against Gram-negative and -positive food-borne pathogens. Films prepared in this manner offer potential as environmentally-friendly active packaging materials for perishable food products. In the future, the application of natural, eco-friendly coatings may become a trend due to consumers’ increasing preferences for high-quality, safe, and cleanly labelled foods. In order to fully verify the applicability of the obtained coatings as packaging materials, further tests should be carried out using model food products. Furthermore, additional research is needed to assess the potential migration of AgNPs into the food matrix.

## Figures and Tables

**Table 1:** Physical properties of double-layered films with plant extracts + AgNPs.

	Control	5% KB	10% KB	15% KB	5% PE	10% PE	15% PE
COLOUR PROPERTIES							
L*	89.78 ^g^ ± 0.23	77.76 ^f^ ± 0.52	66.13 ^e^ ± 0.34	61.89 ^d^ ± 0.86	55.06 ^c^ ± 0.42	41.00 ^b^ ± 0.76	35.52 ^a^ ± 0.24
a*	−1.87 ^a^ ± 0.04	9.17 ^b^ ± 0.23	24.37 ^e^ ± 0.38	26.22 ^f^ ± 0.54	20.51 ^d^ ± 0.10	20.38 ^d^ ± 0.93	14.94 ^c^ ± 0.33
b*	23.86 ^c^ ± 0.94	45.86 ^e^ ± 1.51	55.07 ^f^ ± 0.52	55.12 ^f^ ± 0.66	33.84 ^d^ ± 0.53	21.22 ^b^ ± 1.22	12.93 ^a^ ± 0.34
ΔE	-	27.40	46.30	49.63	23.07	39.91	50.67
Appearance	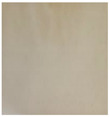	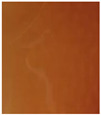	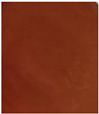	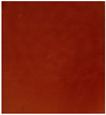	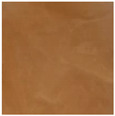	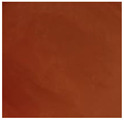	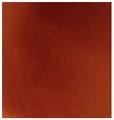
THERMAL PROPERTIES							
Peak temperature (Tm) (°C)	182.8 ^ab^ ± 2.3	178.2 ^a^ ± 4.8	189.3 ^bc^ ± 8.7	191.4 ^c^ ± 2.4	198.4 ^d^ ± 1.7	202.8 ^d^ ± 1.0	205.5 ^d^ ± 0.7
Enthalpy (ΔHm) (J/g)	235.3 ^ab^ ± 1.5	249.1 ^b^ ± 3.9	237.6 ^ab^ ± 6.4	249.5 ^b^ ± 15.1	227.3 ^a^ ± 8.4	237.0 ^ab^ ± 15.7	227.5 ^a^ ± 5.0
WATER PROPERTIES							
WVTR [g/m^2^× d]	840.59 ^a^ ± 48.07	886.9 ^b^ ± 24.63	894.77 ^b^ ± 13.29	921.66 ^bc^ ± 14.94	941.41 ^c^ ± 14.04	967.87 ^c^ ± 18.53	944.42 ^c^ ± 46.29
WCA [°]	CHIT+HGEL	CHIT+HGEL	CHIT+HGEL	CHIT+HGEL	CHIT+HGEL	CHIT+HGEL	CHIT+HGEL
	91.26 ^f^ ± 1.44	88.96 ^e^ ± 0.38	88.27 ^de^ ± 0.64	88.08 ^cd^ ± 0.43	87.46 ^c^ ± 0.77	85.70 ^b^ ± 1.09	81.08 ^a^ ± 1.29
	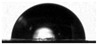	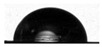	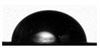	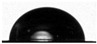	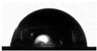	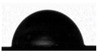	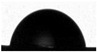
	FUR	FUR + 5% KB	FUR + 10% KB	FUR + 15% KB	FUR + 5% PE	FUR + 10% PE	FUR + 15% PE
	86.34 ^a^ ± 0.23	86.32 ^a^ ± 1.11	87.57 ^b^ ± 1.16	88.98 ^b^ ± 0.39	91.86 ^c^ ± 0.59	94.11 ^d^ ± 0.60	94.68 ^d^ ± 0.70
	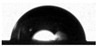	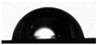	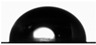	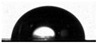	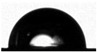	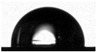	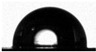
ANTIOXIDANT ACTIVITY							
FRAP iron ion reduction capacity (mMTrolox/mg)	1.55 ^a^ ± 0.29	2.37 ^b^ ± 0.22	5.71 ^d^ ± 0.34	8.32 ^f^ ± 0.78	2.86 ^b^ ± 0.44	3.92 ^c^ ± 0.18	6.87 ^e^ ± 0.68

* Values are expressed as mean ± SD. Different lettering (a–g) in the same rows indicates significant differences (*p* < 0.05).

**Table 2:** Antimicrobial effect of double-layered films with AgNPs.

	10% PE	15% PE	10% KB	15% KB
*Staphylococcus aureus*	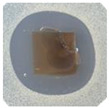	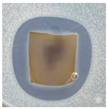	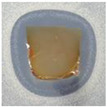	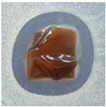
	very good effect with broad inhibition zone ZOI *-20 mm	very good effect with broad inhibition zone ZOI-16 mm	very good effect with broad inhibition zone ZOI-17 mm	very good effect with broad inhibition zone ZOI-17 mm
*Escherichia coli*	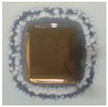	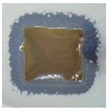	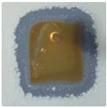	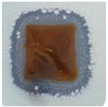
	very good effect with colony growth inside inhibition zone ZOI-17 mm (13 mm internal zone)	very good effect with colony growth inside inhibition zone ZOI-16 mm	very good effect with colony growth inside inhibition zone ZOI-16 mm	very good effect with colony growth inside inhibition zone ZOI-16 mm
*Salmonella enterica*	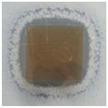	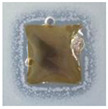	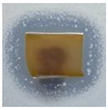	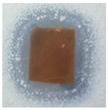
	very good effect with colony growth inside inhibition zone ZOI-17 mm (13 mm internal zone)	very good effect with colony growth inside inhibition zone ZOI-16 mm (13 mm internal zone)	very good effect with colony growth inside inhibition zone ZOI-17 mm	very good effect with colony growth inside inhibition zone ZOI-17 mm (14 mm internal)
*Enterococcus faecalis*	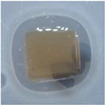	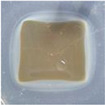	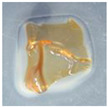	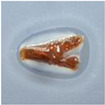
	very good effect with broad inhibition zone ZOI-16 mm	very good effect with narrow inhibition zone ZOI-14 mm	very good effect with narrow inhibition zone ZOI-13 mm	very good effect with narrow inhibition zone ZOI-13 mm
*Pseudomonas aeruginosa*	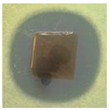	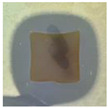	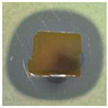	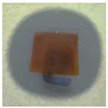
	very good effect with broad inhibition zone ZOI-21 mm	very good effect with broad inhibition zone ZOI-19 mm	very good effect with broad inhibition zone ZOI-20 mm	very good effect with broad inhibition zone ZOI-21 mm
*Candida krusei*	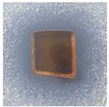	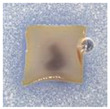	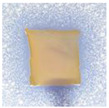	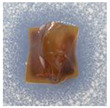
	very good effect with broad inhibition zone ZOI-17 mm	very good effect with narrow inhibition zone ZOI-11 mm	very good effect with narrow inhibition zone ZOI-12 mm	very good effect with broad inhibition zone ZOI-15 mm

* ZOI—zone of inhibition.

## Data Availability

Not applicable.
